# Interspecific association of herbaceous plant communities on different slope orientations and at different altitudes in central Yunnan grasslands

**DOI:** 10.3389/fpls.2024.1461576

**Published:** 2024-12-16

**Authors:** Rui Gong, He-de Gong

**Affiliations:** ^1^ Southwest Forestry University, College of Soil and Water Conservation, Kunming, Yunna, China; ^2^ Qilu Normal University, School of Geography and Tourism, Jinan, Shandong, China

**Keywords:** interspecific association, slope aspect, altitude, χ^2^-test, rank correlation analysis

## Abstract

**Aims:**

Understanding the response of herbaceous plants to habitat changes and the mechanisms of vegetation succession is crucial to the theoretical foundation of the conservation of local vegetation.

**Methods:**

Plots were established at elevations of 1900-2200m, 2200-2500m, and 2500-2800m on both shady and sunny slopes. Four statistical methods 2×2 contingency table χ^2^-test, Spearman's rank correlation coefficient, AC joint coefficient, 17 and Ochiai Index, were employed to analyze the species composition and interspecific associations within each elevation band and aspect.

**Important findings:**

(1) the number of herbaceous plant species was greater on the sunny slope than on the shady slope; the number of species was higher in the2 elevation bands of 1900-2200m and 2200-2500m than in 2500-2800m. (2) Both AC joint coefficient and Ochiai Index revealed that the interspecific connectivity increased as elevation increased on the shady slope, although the highest interspecific connectivity was observed in the 2200-2500m elevation rather than other two elevations on the sunny slope. (3) Negative associations among species pairs were more prevalent than positive associations on both the shady and sunny slopes at all elevations,indicating a high level of negative interspecific associations and connectivity. (4) χ^2^-test values and Spearman rank correlation analysis indicated that it was a relatively unstable community.However, an overall more stable community on the shady slope.The influence of altitude and slope orientation on interspecific associations has wide applications in multiple fields. By deeply understanding the role of these environmental factors, scientists, agricultural workers, forestry managers, and protectors can better carry out work in resource management, species conservation, climate change adaptation, and other aspects.

## 1 Introduction

Plant communities are composed of various interacting life forms, that form a foundation for understanding community structure, function, dynamics, and classification (Gao et al., 2022; Guo et al. 2004). Each community represents the coexistence of species under certain conditions, where species depend on each other, compete with each other, and evolve together (Li et al., 2020). Interactions such as mutual dependence, promotion, restriction, and coevolution occur among species in a community, and contribute to its relative stability (Tu et al., 2022; Zhang, 2010). The inter-specific relationships in a community are shaped by direct or indirect interactions, that result in complex ecological networks (Wilson and Nisbet, 1997). Interspecific associations, where species tend to co-occur at specific spatial scales, are often influenced by environmental differences affecting species distribution (Sanjerehei and Rundel, 2020). Positive correlations in interspecific associations under similar environmental conditions suggest mutually beneficial interactions, such as mutualistic symbiosis, while negative correlations indicate species exclusion, such as competition (Zhang and Rong, 2003). Therefore, it is critical to study interactions between species, their composition and community dynamics to understand population ecology (Shao-Lin et al., 1999). And the impact of climate on the interspecific associations of herbaceous plants on the sunny slope and shady slope.

In the Northern Hemisphere, south-facing slopes are typically characterized as standard sunny slopes, while north-facing slopes are characterized as standard shady slopes (Shao-Lin et al., 1999). These different slope aspects lead to significant climatic differences, that influence soil and vegetation (Lin and Li, 1985). At the same altitude in the middle and low-altitude mountains of southern Shaanxi, studies have shown that soil moisture and biodiversity indices vary between shady and sunny slopes (Cong et al., 2023). Research on the alpine grasslands of Mira Mountain has revealed that the sunny slope has better physical surface soil properties (Yu et al., 2023). Under forested conditions, trees on shady slopes have been found to grow better, consume more water, and be more susceptible to drought fluctuations than trees on sunny slopes (Liu et al., 2022). Another study suggested that shady slopes at a moderate altitude had optimal species richness, aboveground biomass, and soil moisture conditions (Liu et al., 2021).

Different altitudes also affect climatic conditions and influence vegetation. Studies have shown that altitude can alter interspecific associations among tree species (Jin et al., 2022). Factors such as altitude and rock exposure significantly determine the spatial distribution patterns and interspecific associations of karst forest tree species (Zhang et al., 2010). In one study, species pairs showed stronger positive correlations than negative correlations at an altitude of 1800m to 2200m in the eastern part of the Tianshan Mountains, but in the western part the positive correlations were weaker than the negative correlations (Zhang and Zhu, 2015). The strength of interspecific associations was found to be highly dependent on habitat; they generally decreased as altitude increased (habitat type change) (Jiang et al., 2022). The 1800-2000m altitude range was identified as the most suitable area for the concentrated distribution of Stipa grandis in the Tianshan mountain area, but community stability was found to gradually weaken as altitude increased (Zhang et al., 2017).

Historically, research predominantly focused on exploring the correlation between different slope aspects and factors such as biodiversity (Zhang et al., 2019) and soil properties (Huang et al., 2015). Studies also delved into the impact of elevation as a single factor on interspecific associations. However, there has been a notable scarcity in comprehensive investigations regarding the combined influence of both slope aspect and elevation as bivariate variables on interspecific associations. To ensure the rigor of experimental results, this paper selected the slope aspect (sunlit or shaded) in the Dian Central Meadow as the variable for analyzing the influence of slope aspect on interspecific associations. Certain slope aspects, like southeast and northeast, were excluded to allow for a more rigorous contrast in the impact of slope aspect differences between sunlit and shaded slopes on interspecific associations.

This study thus conducted an analysis of interspecific connectivity among major dominant species across different elevation zones of sunlit and shaded slopes in the Dian Central Meadow to explore the effects of varying slope aspects and elevations on the interspecific relationships of dominant species and to analyze the distribution and interspecific associations of these major species within the community. By investigating the influence of changes in slope aspect and elevation on interspecific associations of dominant species, this study aimed to deepen our understanding of how environmental gradients affect ecosystem structure. Recognizing the crucial roles of ecosystem composition and structures in understanding ecological functions and processes is imperative, especially under diverse conditions.

The examination of species distributions and interactions across different slope aspects and elevations contributes to our understanding of their adaptability. This helps reveal species survival strategies under varying environmental conditions. Furthermore, such research provides a foundation for ecological conservation and management and can facilitate the implementation of increasingly effective conservation measures, thereby sustaining ecological balance and safeguarding species diversity.

## 2 Materials and methods

### 2.1 Site description

The research site was located in Xundian Hui and Yi Autonomous County, situated between 102°41′ to 103°33′ east longitude and 25°20′ to 26°01′ north latitude, in the northeast of Kunming City, spanning the watershed between the Jinsha River and Nanpan River basins. To the east it is bordered by Malong County, Zhanyi County, and Huize County, which are connected via roads crossing mountains. To the west it shares boundaries with Fumin County and Luquan County. To the north it is adjacent to Dongchuan and Huize County, which are connected by the Dongchuan Railway, and to the south it borders Songming County, which extends onto the Sichuan-Yunnan plateau. The total land area of Xundian County is 3598 square kilometers, with a longitudinal distance from east to west of over 80 kilometers and a latitudinal distance from south to north of over 60 kilometers. Xundian County has a varied terrain, and there is great variation in altitude. It is characterized by a low-latitude plateau monsoon climate. Winter and spring are predominantly influenced by the westerly circulation, which contribute to a continental monsoon climate with low rainfall and drought. Summer and autumn are mainly controlled by warm and moist air currents from the southwest Pacific or southeast Indian Ocean and are characterized by a prominent maritime monsoon with abundant rainfall and cool, humid conditions. The rainy season lasts from May to October, and the dry season conversely spans November to April of the following year. Two notable characteristics of the climate are 1) significant differences between the valley and mountain areas, and 2) distinct seasons throughout the year. The study area included Hekou Town, Tangdian Town, Jijie Town, Xianfeng Town, and Gongshan Town in Xundian County.

### 2.2 Plot Setup

**Figure 1:**
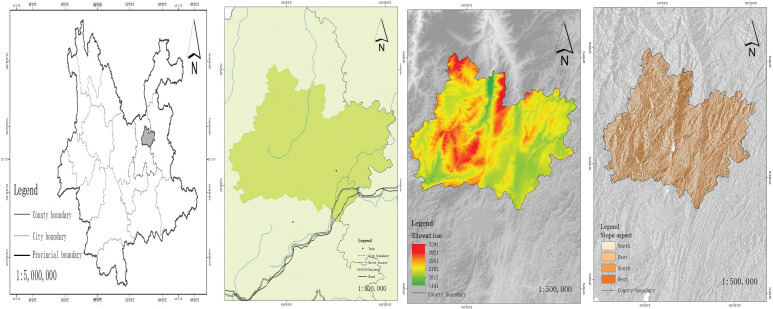
Map of Xundian County.

A total of 16,318 survey points were set up on the shady and sunny slopes (Kuželová and Chytrý, 2004). After screening, survey points suitable for this study were selected on the shady and sunny slopes within the altitudinal ranges of 1900-2200m, 2200-2500m, and 2500-2800m. Survey points were set up at intervals of 300m within the altitudinal range of 1900-2800m on the shady and sunny slopes, with 8169 grassland survey points set up on each slope. The altitude difference between the lowest and highest altitude plots on the shady slope was 839m, and on the sunny slope it was 893m. The slope direction and altitude profiles of the shady and sunny slopes are shown in **Figure 1**.

Due to the diverse range of plant species on shady and sunny slopes, each plant was sorted based on its frequency of occurrence in the plots, in descending order. For ease of calculation, only the most predominant herbaceous plants, those with higher occurrence frequencies, were selected for inclusion in interspecific association analysis. In the analysis, 23 herbaceous plant species were selected from the shady slope and 31 herbaceous plant species from the sunny slope, 35 species in total as shown in **Table 1**.

**Table 1:** Herbaceous Plant Species and Codes.

Code	Species Name	Family Name
1	Artemisia selengensis	*Asteraceae*
2	Pteridium aquilinum	*Dennstaedtiaceae*
3	Imperata cylindrica	*Poaceae*
4	Arundinella hirta	*Poaceae*
5	Artemisia caruifolia	*Asteraceae*
6	Miscanthus sinensis	*Poaceae*
7	Arundinella hookeri	*Poaceae*
8	Carex breviculmis	*Cyperaceae*
9	Trifolium repens	*Fabaceae*
10	Ageratina adenophora	*Asteraceae*
11	Cynodon dactylon	*Poaceae*
12	Eragrostis pilosa	*Poaceae*
13	Schizachyrium delavayi	*Poaceae*
14	Saccharum rufipilum	*Poaceae*
15	Bromus catharticus	*Poaceae*
16	Bidens pilosa	*Asteraceae*
17	Capillipedium parviflorum	*Poaceae*
18	Lolium perenne	*Poaceae*
19	Poa annua	*Poaceae*
20	Eleusine indica	*Poaceae*
21	Centella asiatica	*Apiaceae*
22	Argentina lineata	*Rosaceae*
23	Picris hieracioides	*Asteraceae*
24	Oxyria sinensis	*Polygonaceae*
25	Festuca ovina	*Poaceae*
26	Bromus japonicus	*Poaceae*
27	Dactylis glomerata	*Poaceae*
28	Fragaria vesca	*Rosaceae*
29	Artemisia viscida	*Asteraceae*
30	Eremochloa ophiuroides	*Poaceae*
31	Eulaliopsis binata	*Poaceae*
32	Potentilla chinensis	*Rosaceae*
33	Agrimonia pilosa	*Rosaceae*
34	Polypogon fugax	*Poaceae*
35	Paspalum thunbergii	*Poaceae*

## 3 Data analysis

### 3.1 Test of interspecific association

In this study, we selected a total of 35 dominant herbaceous plant species along the elevational gradient of the shady and sunny slopes in Xundian County for analysis and calculation. Following the method proposed by Wang and Peng (1985) and others, a 2×2 contingency table was constructed, and the values (a, b, c, d) for each species pair were calculated (**Table 2**).

**Table 2:** 2×2 contingency table.

Species		Species B		Sum
		Present	Absent	
Species A	Present	a	b	a+b
	Absent	c	d	c+d
	Sum	a+c	b+d	a+b+c+d

To assess the accuracy of the association between species, this study selected the χ² statistic to test the association between species. Because the experimental samples were non-continuous, the χ²-value was calculated using the Yates continuity correction formula (Cox, 1980; Chai et al., 2016).


(1) $$\chi^{2}=\frac{\text{N}{\left(\left|\text{ad}-\text{bc}\right|-\frac{N}{2}\right)}^{2}}{\left(a+b\right)\left(c+d\right)\left(a+c\right)\left(b+d\right)}$$

where n represents the total sample size; a denotes the number of sample points where both species occur; b and c refer to the sample points where only one species occurs; d stands for the number of sample points where neither species occurs.

When V = ad − bc > 0, this indicates a positive association, while V< 0 suggests a negative association. For a 2×2 contingency table, there is 1 degree of freedom. If 3.841< χ²< 6.635 (0.01< P< 0.05), this indicates a significant association between species pairs. If χ² > 6.635 (P< 0.01), this denotes a highly significant association. Otherwise, the association is not significant.

### 3.2 Spearman's rank correlation coefficient test


(2) $$R_{s}\left(i,j\right)=1-\frac{6\sum_{q=1}^{N}D_{q}^{\;2}}{N^{3}-N}$$

where R represents the correlation coefficient between i and j (Nguyen et al., 2023), N is the number of samples, *D_q_* denotes the rank difference for each species, and *K_iq_* and *K_jq_* respectively represent the values of species *i* and species *j* in sample *q*, the calculation for *D_q_* is: [ *D_q_* = (*K_iq_* - *K_jq_*) ]

### 3.3 Inter-species association coefficient

Although the χ²-value indicates the degree of association between species pairs and provides a qualitative insight, it does not quantitatively reflect the magnitude and direction of species associations. Therefore, it was necessary to introduce the association coefficient (AC) to fill this gap (Chun-nan et al., 2013; Hurlbert, 1969). The calculation is illustrated in Formulas 3 to 5.


(3) $$\text{AC}=\frac{ad-bc}{\left(a+b\right)\left(b+d\right)}\;(\text{ad>bc})$$

(4) $$\text{AC}=\frac{ad-bc}{\left(a+b\right)\left(a+c\right)}\;(\text{ad≤bc},\;\text{d≥a})$$

(5) $$\text{AC}=\frac{ad-bc}{\left(b+d\right)\left(c+d\right)}\;(\text{ad≤bc},\;\text{d≤a})$$

The value of AC always falls within the range of [-1, 1]. A higher AC value near to 1 indicates that one species has a clear advantage or that both species have highly similar habitat requirements. Conversely, a lower AC value suggests a certain degree of inhibition or direct exclusion between the two species. The sign of the difference between ad and bc determines the sign of AC. If (ad-bc>0), the two species are positively correlated; if (ad-bc<0), they are negatively correlated; and if (ad-bc=0), there is no association, and the species are most likely independent of each other. Notably, when (bc=0), this is considered the maximum positive correlation, while (ad=0) represents the maximum negative correlation.

### 3.4 Interspecies association determination

AC can determine the magnitude and direction of association, but it is heavily influenced by the value of ( d ), which leads to some bias in the results. To address this issue, the Inter-Species Association Index, the OI (Ochiai Index), was introduced for calibration (Reynolds, 1988; Zhou et al., 2000).


(6) $$\text{OI}=\frac{a}{\sqrt{(a+b)(a+c)}}$$

The higher the value of the association index, the greater the probability of simultaneous occurrence of species. However, a high value does not necessarily indicate positive correlation. An index value of 0 means there is "no association," which indicates complete dissimilarity between species and that species do not occur together in the same sample. When there is "maximum association," the index value is 1. Including the OI provides some degree of correction for the biases in the AC.

### 3.5 Data processing

After preliminary organization in Excel 2007, the data underwent further analysis using SPSS26 and The R Programming Language. Additionally, OriginPro 2021 was employed for data visualization.

## 4 Results and analysis

### 4.1 Overview of herbaceous plant families and genera composition within plots

Across two slopes facing different aspects (north and south), three altitudinal bands were established, ranging from 1900 to 2200m, 2200 to 2500m, and 2500 to 2800m. A total of 16,318 plots were set up, encompassing 35 main species. These species represented eight major families; *Poaceae* (57.1%), *Asteraceae* (17.1%), *Rosaceae* (11.4%), *Fabaceae* (2.9%), *Dennstaedtiaceae* (2.9%), *Cyperaceae* (2.9%), *Apiaceae* (2.9%), and *Polygonaceae* (2.9%). *Poaceae* represented the largest proportion of herbaceous plants.

On the north-facing slope, within the elevation band of 1900-2200m, there were 17 dominant species. The elevation band of 2200-2500m had 13 dominant species, and the elevation band of 2500-2800m had 14 dominant species. On the south-facing slope, within the elevation band of 1900-2200m, there were 19 dominant species. The elevation band of 2200-2500m had 20 dominant species, and the elevation band of 2500-2800m had 12 dominant species.

### 4.2 χ^2^test

**Figure 2:**
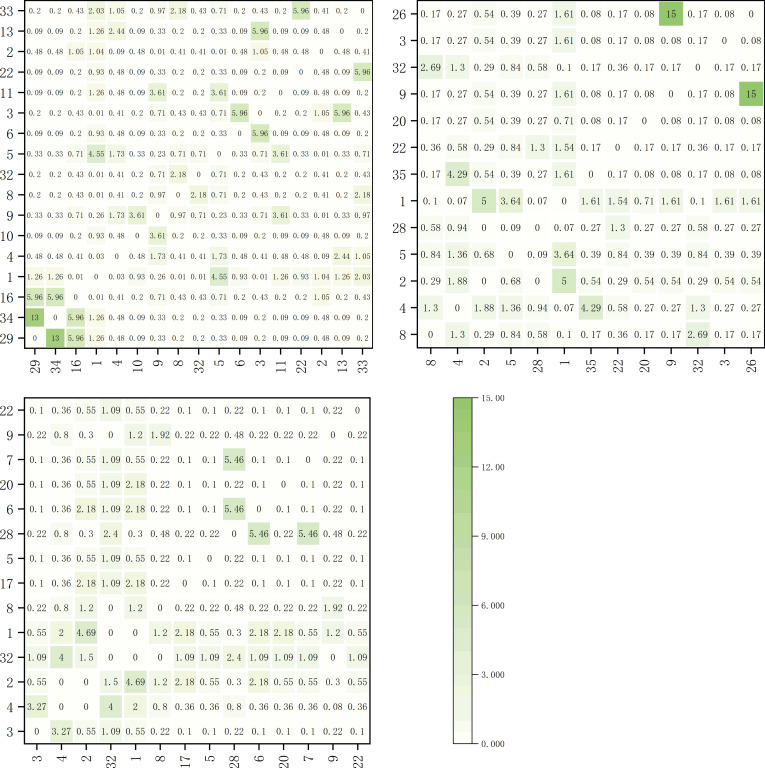
Chi-squared value matrix map of major herbaceous plants on shady slopes within elevation bands of 1900-2200m, 2200-2500m, and 2500-2800m.

The chi-squared values of dominant herbaceous plant species on the shady slope are shown in **Figure 2**. Within the elevation band of 1900-2200m, there were 17 dominant species forming a total of 136 species pairs. Among them, one pair showed extremely significant correlation, *Polypogon fugax* and *Artemisia viscida*, and six pairs showed significant correlation, *Bidens pilosa* and *Artemisia viscida*, *Bidens pilosa* and *Polypogon fugax*, *Artemisia selengensis* and *Artemisia caruifolia*, *Miscanthus sinensis* and *Imperata cylindrica*, *Imperata cylindrica* and *Schizachyrium delavay*, and *Argentina lineata* and *Agrimonia pilosa*.

Within the elevation band of 2200-2500m, there were 13 dominant species forming a total of 78 species pairs. Among them, one pair showed extremely significant correlation, *Trifolium repens* and *Bromus japonicus*, and two pairs showed significant correlation, *Paspalum thunbergii* and *Arundinella hirta*, and *Pteridium aquilinum* and *Artemisia selengensis*.

Within the elevation band of 2500-2800m, there were 14 dominant species forming a total of 91 species pairs. Among them, four pairs showed significant correlation, *Arundinella hirta* and *Potentilla chinensis*, *Pteridium aquilinum* and *rtemisia selengensis*, *Fragaria vesca* and *Miscanthus sinensis*, and *Fragaria vesca* and *Arundinella hookeri*.

**Figure 3:**
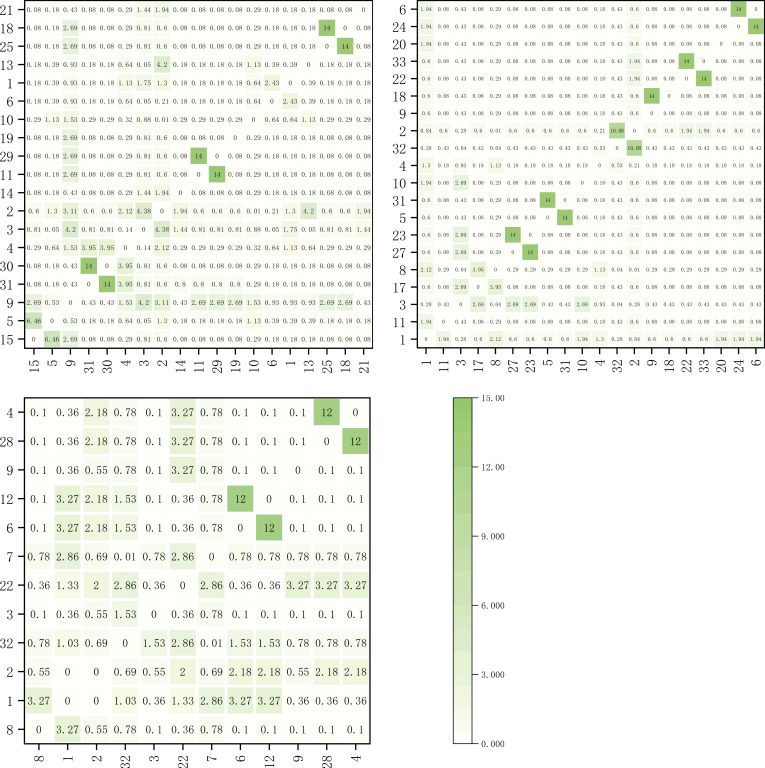
Chi-squared value matrix map of major herbaceous plants on sunny slopes within elevation bands of 1900-2200m, 2200-2500m, and 2500-2800m.

The chi-squared values for dominant herbaceous plant species on the sunny slope are shown in **Figure 3**. Within the elevation band of 1900-2200m, there were 19 dominant species forming a total of 171 species pairs. Notably, three pairs exhibited an extremely significant correlation: *Eulaliopsis binata* and *Eremochloa ophiuroides*, *Cynodon dactylon* and *Artemisia viscida*, and *Festuca ovina* and *Lolium perenne*. Additionally, six pairs showed significant correlation: *Bromus catharticu*s and *Artemisia caruifolia*, *Trifolium repens* and *Imperata cylindrica*, *Eulaliopsis binata* and *Arundinella hirta*, *Eremochloa ophiuroides* and *Arundinella hirta*, *Imperata cylindrica* and *Pteridium aquilinum*, and *Pteridium aquilinum* and *Schizachyrium delavayi*.

Within the elevation band of 2200-2500m, there were 20 dominant species of herbaceous plants forming a total of 190 species pairs. Among them, six pairs exhibited an extremely significant correlation: *Picris hieracioides* and *Dactylis glomerata*, *Artemisia caruifolia* and *Eulaliopsis binata*, *Potentilla chinensis* and *Pteridium aquilinum*, *Trifolium repens* and *Lolium perenne*, *Argentina lineata* and *Agrimonia pilosa*, *Oxyria sinensis* and *Miscanthus sinensis*. Additionally, one pair showed significant correlation, *Capillipedium parviflorum* and *Carex breviculmis.*

Within the elevation band of 2500-2800m, there were 12 dominant species of herbaceous plants forming a total of 66 species pairs. Among them, two pairs exhibited an extremely significant correlation: *Miscanthus sinensis* and *Eragrostis pilosa*, *Fragaria vesca* and *Arundinella hirta*.

As shown in **Table 3**, as the elevation bands on the shaded slope increased in altitude, there were significant and highly significant correlations observed for species proportions. With proportions of 4.47%, 3.9%, and 4.4%, the species showed a trend of initially decreasing and then increasing as the elevation bands increased in altitude. On the sunny slope, there were similarly significant and highly significant correlations observed for species proportions as the elevation bands increased in altitude, though with proportions of 5.3%, 3.25%, and 3.0%, the trend showed a pattern of decrease.

**Table 3:** Analysis of χ2 test values for different slope aspects in different elevation bands.

Slope aspect	Altitude	Total species logarithm	Scope	Logarithmic number of species	Proportion (%)
Shady slope	1900	136	χ^2^<3.814	129	94.9
			3.814≤χ^2^≤6.635	6	4.4
			χ^2^>6.635	1	0.07
	2200	78	χ^2^<3.814	75	96.2
			3.814≤χ^2^≤6.635	2	2.6
			χ^2^>6.635	1	1.3
	2500	91	χ^2^<3.814	87	95.6
			3.814≤χ^2^≤6.635	4	4.4
			χ^2^>6.635	0	0
Sunny slope	1900	171	χ^2^<3.814	162	94.7
			3.814≤χ^2^≤6.635	6	3.5
			χ^2^>6.635	3	1.8
	2200	190	χ^2^<3.814	183	96.3
			3.814≤χ^2^≤6.635	1	0.05
			χ^2^>6.635	6	3.2
	2500	66	χ^2^<3.814	64	97.0
			3.814≤χ^2^≤6.635	0	0
			χ^2^>6.635	2	3.0

Between 1900-2200m, the sunny slope exhibited a higher proportion of significantly and highly significantly correlated species than did the shaded slope. However, between 2200-2500m and 2500-2800m, the shaded slope showed a higher proportion of significantly and highly significantly correlated species than did the sunny slope.

### 4.3 Spearman's rank correlation coefficient

**Figure 4:**
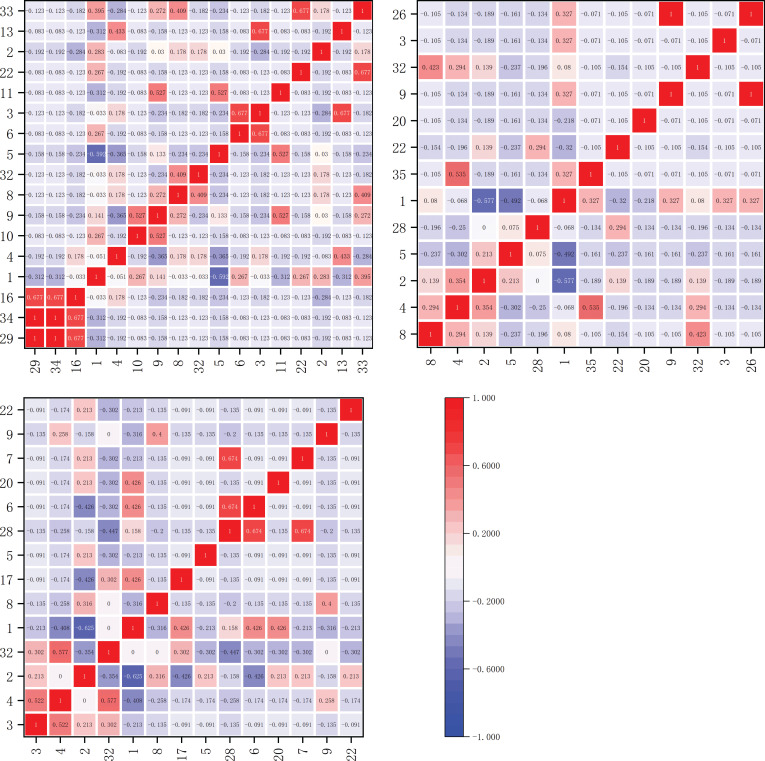
Spearman rank correlation coefficient matrix for main herbaceous plants on the shady slope within elevation bands of 1900-2200m, 2200-2500m, and 2500-2800m.

The Spearman correlation coefficients for dominant herbaceous species on the shaded slope are shown in **Figure 4**. Within the elevation band of 1900-2200m, there were 17 dominant species forming a total of 136 species pairs. Among these pairs, 29 pairs correlated positively and 107 pairs correlated negatively, accounting for 21.3% and 78.7% of the total species pairs, respectively. Notably, there were 8 significantly positively correlated pairs, *Artemisia viscida* with *Bidens pilosa*, *Polypogon fugax* with *Bidens pilosa*, *Ageratina adenophora* with *Trifolium repens*, *Trifolium repens* with *Cynodon dactylon*, *Artemisia caruifolia* with *Cynodon dactylon*, *Miscanthus sinensis* with *Imperata cylindrica*, *Imperata cylindrica* with *Schizachyrium delavayi*, and *Argentina lineata* with *Agrimonia pilosa*. Additionally, one pair exhibited a highly significant positive correlation, *Artemisia viscida* with *Polypogon fugax*. Furthermore, there was 1 significantly negatively correlated pair, *Artemisia caruifolia* with *Artemisia selengensis*.

Within the elevation band of 2200-2500m, there were 13 dominant species forming a total of 78 species pairs. Among these pairs, 18 pairs correlated positively, 59 pairs correlated negatively, and 1 pair was uncorrelated, accounting for 23.1%, 75.6%, and 1.3% of the total species pairs, respectively. Notably, there was 1 significantly positively correlated pair, *Arundinella hirta* with *Paspalum thunbergii*, and 1 highly significant positively correlated pair, *Trifolium repens* with *Bromus japonicus*. Additionally, there was 1 significantly negatively correlated pair, *Artemisia selengensis* with *Pteridium aquilinum*, and 1 uncorrelated pair, *Pteridium aquilinum* with *Fragaria vesca*.

Within the elevation band of 2500-2800m, there were 14 dominant species forming a total of 91 species pairs. Among these pairs, 17 pairs correlated positively, 70 pairs correlated negatively, and 4 pairs were uncorrelated, accounting for 18.7%, 76.9%, and 4.4% of the total species pairs, respectively. Notably, there were 4 significantly positively correlated pairs, *Imperata cylindrica* with *Arundinella hirta*, *Arundinella hirta* with *Potentilla chinensis*, *Fragaria vesca* with *Miscanthus sinensis*, and *Fragaria vesca* with *Arundinella hookeri*. There was also 1 significantly negatively correlated pair, *Pteridium aquilinum* with *Artemisia selengensis*. Additionally, there were four uncorrelated pairs, *Pteridium aquilinum* with *Artemisia selengensis*, *Potentilla chinensis* with *Carex breviculmis*, *Potentilla chinensis* with *Trifolium repens*, and *Arundinella hirta* with *Pteridium aquilinum.*

**Figure 5:**
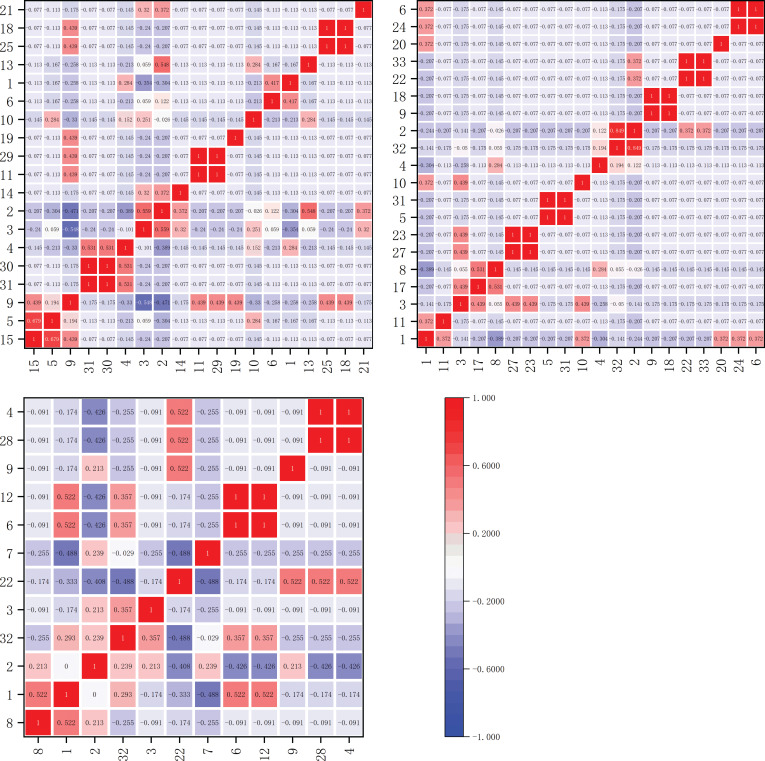
Spearman rank correlation coefficient matrix for main herbaceous plants on the sunny slope within elevation bands of 1900-2200m, 2200-2500m, and 2500-2800m.

The Spearman rank correlation coefficients for dominant herbaceous species on the sunny slope are illustrated in **Figure 5**. Within the elevation band of 1900-2200m, there were 19 dominant species forming a total of 171 species pairs. Among these pairs, 27 pairs correlated positively and 144 pairs correlated negatively, accounting for 15.8% and 84.2% of the total species pairs, respectively. Notably, there were 5 significantly positively correlated species pairs, *Bromus catharticu*s with *Artemisia caruifolia*, *Eulaliopsis binata* with *Arundinella hirta*, *Eremochloa ophiuroides* with *Arundinella hirta*, *Imperata cylindrica* with *Pteridium aquilinum*, and *Pteridium aquilinum* with *Schizachyrium delavayi*. Additionally, there was 1 highly significant positively correlated species pair, *Cynodon dactylon* with *Cynodon dactylon*. Furthermore, there was 1 significantly negatively correlated species pair, *Trifolium repens* with *Imperata cylindrica*.

Within the elevation band of 2200-2500m, there were 20 dominant species forming a total of 190 species pairs. Among these pairs, 23 pairs correlated positively and 167 pairs correlated negatively, accounting for 12.1% and 87.9% of the total species pairs, respectively. Notably, there were 2 significantly positively correlated species pairs, *Capillipedium parviflorum* with *Carex breviculmis* and *Potentilla chinensis* with *Pteridium aquilinum*. Additionally, there were 5 highly significant positively correlated species pairs, *Dactylis glomerata* with *Picris hieracioides*, *Artemisia caruifolia* with *Eulaliopsis binata*, *Trifolium repens* with *Lolium perenne*, *Argentina lineata* with *Agrimonia pilosa*, and *Oxyria sinensis* with *Miscanthus sinensis*.

Within the elevation band of 2500-2800m, there were 12 dominant species forming a total of 66 species pairs. Among these pairs, 17 pairs correlated positively, 48 pairs correlated negatively, and 1 pair was uncorrelated, accounting for 30.9%, 67.3%, and 1.8% of the total species pairs, respectively. Notably, there were 6 significantly positively correlated pairs, *Carex breviculmis* with *Artemisia selengensis*, *Artemisia selengensis* with *Miscanthus sinensis*, *Artemisia selengensis* with *Eragrostis pilosa*, *Argentina lineata* with *Trifolium repens*, *Argentina lineata* with *Fragaria vesca*, and *Argentina lineata* with *Arundinella hirta*. Additionally, there were 2 highly significant positively correlated pairs, *Miscanthus sinensis* with *Eragrostis pilosa* and *Fragaria vesca* with *Arundinella hirta*. Finally, there was 1 uncorrelated pair, *Artemisia selengensis* with *Pteridium aquilinum*.

As shown in **Table 4**, within different elevation bands on both the shady and sunny slopes, there were a higher number of species with negative correlations than with positive correlations. This suggests the presence of some competition or exclusion phenomena in the community. The positive-to-negative correlation ratios for shady slopes were 0.27, 0.31, and 0.24 at the different altitudes, in order of increasing elevation, respectively. These results show a trend of initial increasing and subsequent decrease as altitude increased. For sunny slopes the positive-to-negative correlation ratios were 0.19, 0.14, and 0.35 at the different altitudes, in order of increasing elevation, respectively. These results show a trend of initial decrease and then subsequent increase as altitude increased.

**Table 4:** Spearman's rank correlation coefficient analysis for different slopes and elevation bands, ++ represents extremely significant correlation, + represents significant correlation, — indicates no significant correlation, and T represents total count.

Slope aspect	Altitude	Positive association				Negative association				Positive to negative ratio
		++	+	—	T	++	+	—	T	
Shady slope	1900	1	8	20	29	0	1	106	107	0.27
	2200	1	1	16	18	0	1	58	59	0.31
	2500	0	4	13	17	0	1	69	70	0.24
Sunny slope	1900	1	5	21	27	0	1	143	144	0.19
	2200	5	2	16	23	0	0	167	167	0.14
	2500	2	6	9	17	0	0	48	48	0.35

The positive-to-negative correlation ratios were greater on shady slopes than sunny slopes at 1900-2200m and 2200-2500m. However, at 2500-2800m, the positive-to-negative correlation ratio was greater on sunny slopes than on shady slopes.

### 4.4 Interspecific association coefficient

**Figure 6:**
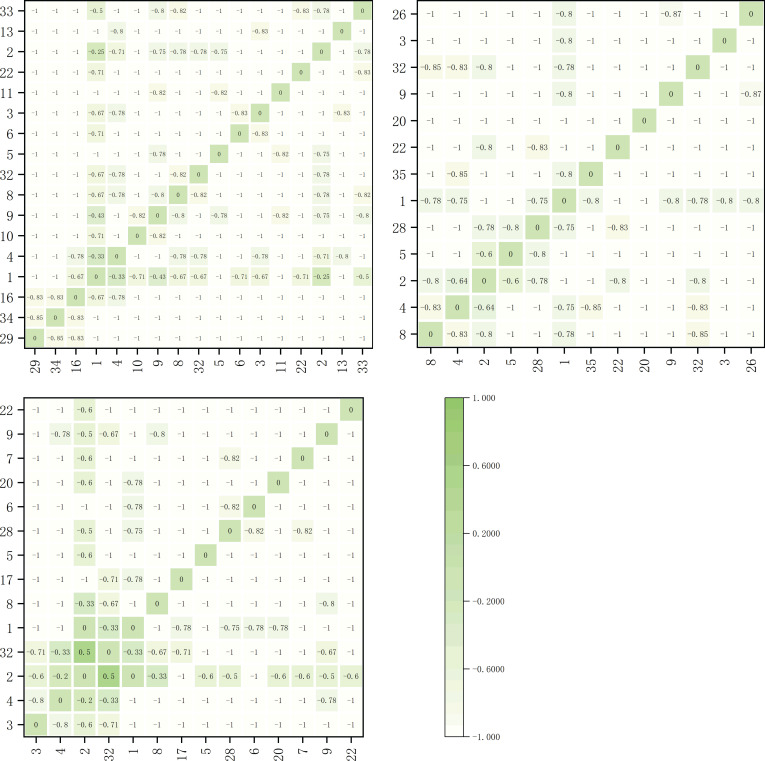
Matrix of the inter-specific Association Coefficient (AC) for the main herbaceous plants on shady slopes within elevation bands of 1900-2200m, 2200-2500m, and 2500-2800m.

The interspecific association coefficients (AC) among dominant herbaceous plant species on the shady slope are depicted in **Figure 6**. The AC values determined whether the associations between species were positive or negative. Within the elevation band of 1900-2200m, there were 17 dominant species forming a total of 136 species pairs. Among these, there were 8 significantly negatively correlated pairs and 128 extremely significantly negatively correlated pairs. Within the elevation band of 2200-2500m, there were 13 dominant species forming a total of 78 species pairs. Among these, there were 2 significantly negatively correlated pairs and 76 extremely significantly negatively correlated pairs. Within the elevation band of 2500-2800m, there were 14 dominant species forming a total of 91 species pairs. Among these, there was 1 significantly positively correlated pair, *Pteridium aquilinum* and *Potentilla chinensis*. There were 9 significantly negatively correlated pairs and 76 extremely significantly negatively correlated pairs.

**Figure 7:**
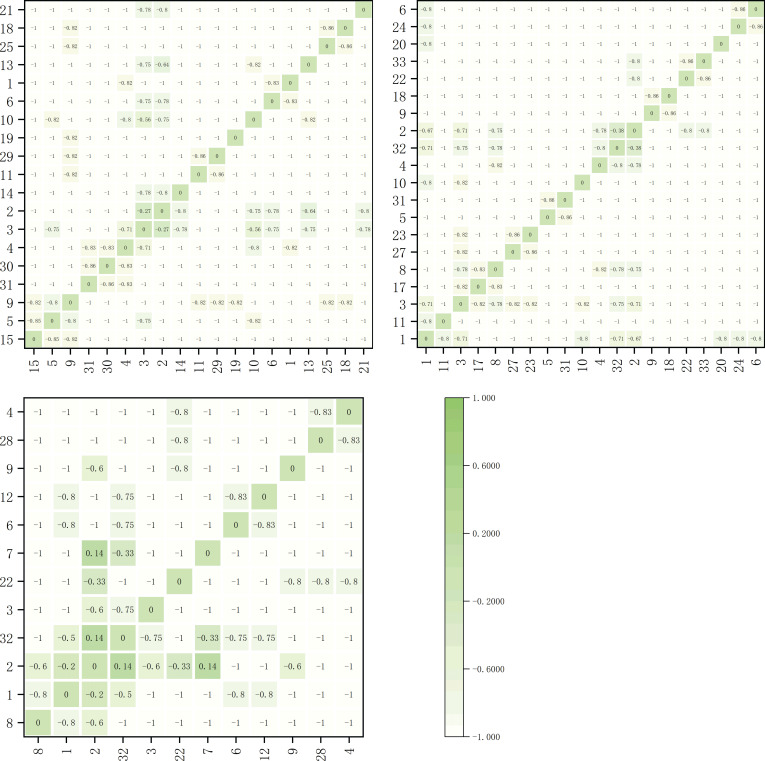
Matrix of the inter-specific Association Coefficient (AC) for the main herbaceous plants on sunny slopes within elevation bands of 1900-2200m, 2200-2500m, and 2500-2800m.

The inter-specific association coefficients (AC) among dominant herbaceous plant species on the sunny slope are depicted in **Figure 7**. The AC values determined whether the associations between species were positive or negative. Within the elevation band of 1900-2200m, there were 19 dominant species forming a total of 171 species pairs. Among these, there were 3 significantly negatively correlated pairs and 168 extremely significantly negatively correlated pairs. Within the elevation band of 2200-2500m, there were 20 dominant species forming a total of 190 species pairs. Among these, there were 2 significantly negatively correlated pairs and 188 extremely significantly negatively correlated pairs. Within the elevation band of 2500-2800m, there were 12 dominant species forming a total of 66 species pairs. Among these, there were 2 significantly positively correlated pairs, *Pteridium aquilinum* and *Potentilla chinensis*, and *Pteridium aquilinum* and *Arundinella hookeri*. There were 4 significantly negatively correlated pairs and 60 extremely significantly negatively correlated pairs.

From the AC values, it can be observed that the calculated results were consistent with the overall connectivity analysis results. The number of negatively connected species pairs was generally higher than that of positively connected species pairs, with a high prevalence of extremely strong negative connections. This indicates intense competition among species in the community, which likely led to an unstable state. However, positive connections between species pairs were observed within the elevation band of 2500-2800m on both shady and sunny slopes, with significant positive correlations observed on the shady slopes.

### 4.5 Ochiai index

**Figure 8:**
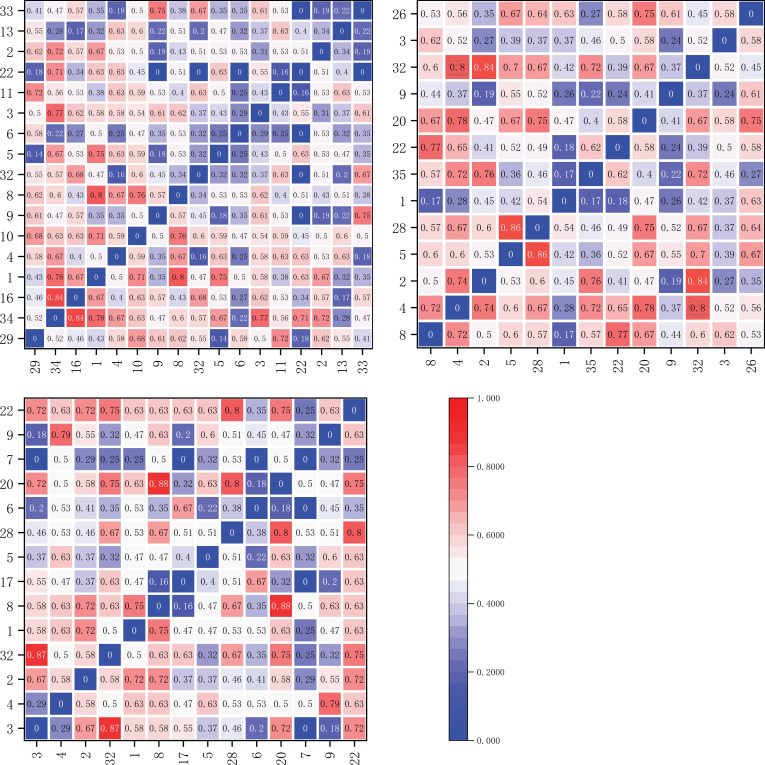
Matrix plot of the Ochiai Index (OI) values for the dominant herbaceous plants on the shady slope within elevation bands of 1900-2200m, 2200-2500m, and 2500-2800m.

The Ochiai Index (OI) values for dominant herbaceous plant species on the shady slope are illustrated in **Figure 8**. Within the elevation band of 1900-2200m, there were 17 dominant species comprising a total of 136 species pairs. The OI values fell within the ranges [0,0.2), [0.2,0.3), [0.3,0.5), and [0.5,1], with 13, 10, 35, and 78 pairs, respectively. These results indicate that 58 species pairs had weak linkage (0≤OI<0.5), and 78 pairs had strong linkage (0.5≤OI ≤ 1).

Within the elevation band of 2200-2500m, there were 13 dominant species forming a total of 78 species pairs. The OI values fell within the ranges [0,0.2), [0.2,0.3), [0.3,0.5), and [0.5,1], with 4, 7, 21, and 46 pairs, respectively. The results indicate that 32 pairs of species had weak linkage (0≤OI<0.5), and 46 pairs had strong linkage (0.5≤OI ≤ 1).

Within the elevation band of 2500-2800m, there were 14 dominant species constituting a total of 91 species pairs. The OI values fell within the ranges [0,0.2), [0.2,0.3), [0.3,0.5), and [0.5,1], with 6, 8, 23, and 54 pairs, respectively. The results indicate that 37 pairs of species had weak linkage (0≤OI<0.5), and 54 pairs had strong linkage (0.5≤OI ≤ 1).

**Figure 9:**
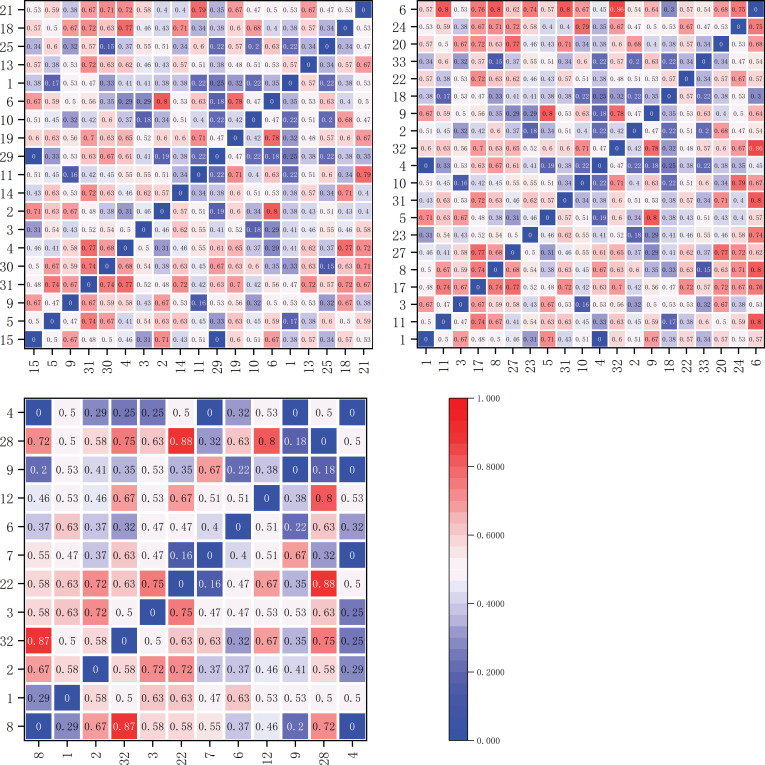
Matrix plot of the Ochiai Index (OI) values for the dominant herbaceous plants on the sunny slope within elevation bands of 1900-2200m, 2200-2500m, and 2500-2800m.

On the sunny slope, the Ochiai Index (OI) values for dominant herbaceous plant species are illustrated in **Figure 9**. Within the elevation band of 1900-2200m, there were 19 dominant species forming a total of 171 species pairs. The OI values fell within the ranges [0,0.2), [0.2,0.3), [0.3,0.5), and [0.5,1], with 7, 10, 61, and 93 pairs, respectively. The results indicate that 78 pairs of species had weak linkage (0≤OI<0.5), and 93 pairs had strong linkage (0.5≤OI ≤ 1).

Within the elevation band of 2200-2500m, there were 20 dominant species forming a total of 190 species pairs. The OI values fell within the ranges [0,0.2), [0.2,0.3), [0.3,0.5), and [0.5,1], with 7, 10, 63, and 110 pairs, respectively. The results indicate that 80 pairs of species had weak linkage (0≤OI<0.5), and 110 pairs had strong linkage (0.5≤OI ≤ 1).

Within the elevation band of 2500-2800m, there were 12 dominant species forming a total of 66 species pairs. The OI values fell within the ranges [0,0.2), [0.2,0.3), [0.3,0.5), and [0.5,1], with 5, 6, 17, and 38 pairs, respectively. The results indicate that 28 pairs of species had weak linkage (0≤OI<0.5), and 38 pairs had strong linkage (0.5≤OI ≤ 1).

As shown in **Table 5**, in increasingly high elevation bands on the shady slope, the proportions of significantly and highly significantly associated species pairs (OI value ≥ 0.5) were 57.4%, 59%, and 59.3%, respectively, demonstrating an increasing trend. Conversely, in increasingly high elevation bands on the sunny slope, the proportions of significantly and highly significantly associated species pairs were 54.4%, 57.9%, and 57.6%, respectively, showing a trend of initial increase and subsequent decrease.

**Table 5:** Analysis of Ochiai Index (OI) values in different slope aspects and elevation bands.

Slope aspect	Altitude	Total species logarithm	Scope	Total logarithm	Proportion
Shady slope	1900	136	0≤OI<0.3	23	16.9
			0.3≤OI<0.5	35	25.7
			0.5≤OI ≤ 1	78	57.4
	2200	78	0≤OI<0.3	11	14.1
			0.3≤OI<0.5	21	26.9
			0.5≤OI ≤ 1	46	59.0
	2500	91	0≤OI<0.3	14	15.4
			0.3≤OI<0.5	23	25.3
			0.5≤OI ≤ 1	54	59.3
Sunny slope	1900	171	0≤OI<0.3	17	9.9
			0.3≤OI<0.5	61	35.7
			0.5≤OI ≤ 1	93	54.4
	2200	190	0≤OI<0.3	17	8.9
			0.3≤OI<0.5	63	33.2
			0.5≤OI ≤ 1	110	57.9
	2500	66	0≤OI<0.3	11	16.7
			0.3≤OI<0.5	17	25.8
			0.5≤OI ≤ 1	38	57.6

Furthermore, the proportions of significantly and highly significantly associated species pairs at each elevation band, 1900-2200m, 2200-2500m, and 2500-2800m, were all higher on the shady slope than on the sunny slope.

## 5 Discussion and conclusion

### 5.1 Discussion

The present study conducted interspecific association analysis by selecting dominant species within different slope aspects and elevation bands, employing four test methods: χ2-test, Spearman's rank correlation coefficient, AC association coefficient, and Ochiai Index. Compared to the χ2-test, the AC association coefficient exhibited a higher proportion of negatively associated species pairs. This phenomenon can be attributed to two main factors. Firstly, the χ2-test is derived from presence-absence data of dominant species on shady and sunny slopes. To some extent, presence-absence data collection weakens interspecific associations and inevitably leads to information loss. Secondly, in the AC association coefficient, when a dominant species is absent within a plot, it amplifies the absence of association between two species, resulting in more negative associations than positive ones (Zhou et al., 2022). Therefore, supplementary use of the Ochiai Index and Spearman's rank correlation coefficient was warranted.

Within the elevation band of 1900-2200m on the shady slope, among 136 species pairs exhibiting a significantly negative correlation in their abundance correlation (AC), 78 pairs displayed a strong association in the OI (Ochiai Index) results. In the elevation band of 2200-2500m, out of 78 species pairs showing a significantly negative AC, 46 pairs exhibited a strong association in the OI results. Similarly, in the elevation band of 2500-2800m, among 91 species pairs with a significantly negative AC, 54 pairs demonstrated a strong association in the OI results. On the sunny slope, within the elevation band of 1900-2200m, out of 171 species pairs with a significantly negative AC, 93 pairs showed a strong association in the OI results. In the elevation band of 2200-2500m, among 190 species pairs exhibiting a significantly negative AC, 110 pairs displayed a strong association in the OI results. Lastly, within the elevation band of 2500-2800m, out of 66 species pairs with a significantly negative AC, 38 pairs exhibited a strong association in the OI results.

These findings suggest a high degree of negative correlation and association among species pairs. The significant negative correlations indicate strong competitive relationships among species, while the strong associations imply ongoing interdependence or coexistence. This suggests a certain level of resource differentiation allowing different species to coexist within the same ecosystem (Greig-Smith, 1983; Freckman and Caswell, 1985). It also indicates the presence of a relatively stable dynamic equilibrium within the community. Despite interspecies competition, their interactions contribute to maintaining the stability of the ecosystem, preventing the extinction of certain species due to excessive competition.

On the shady slope, the ratio of positive to negative Spearman rank correlation coefficients between different elevations initially increased and then decreased as elevation rose. Conversely, on the sunny slope, this ratio initially decreased and then increased with increasing elevation. At elevation bands of 1900-2200m and 2200-2500m, the ratio of positive to negative correlations was greater on the shady slope than on the sunny slope. However, at 2500-2800m, the ratio of positive to negative correlations was greater on the sunny slope than on the shady slope.

Elevation is typically associated with environmental factors such as climate conditions, soil type, and vegetation structure. As elevation increases, there may be more resource limitations, such as decreasing temperatures and deteriorating soil quality, that lead to changes in vegetation composition and species structure. Higher elevations may facilitate the formation of vertically stratified vegetation structures, thereby altering competitive relationships among different plant species. Some alpine plants may adapt to lower temperatures and shorter growing seasons, while other species may dominate in lower elevation areas, thus leading to changes in interspecies associations.

Variation in climate conditions at different elevations significantly impacts the associations among dominant species. For instance, as elevation increases, temperatures tend to decrease, and gradients in precipitation and solar radiation intensity may also occur, thus affecting plant growth and reproduction. Higher species diversity may be observed at lower elevations. This is attributed to changes in climate, soil, and other environmental factors that may also shift the relative abundance of different species across elevation zones.

The research site was located in an area with a subtropical monsoon climate, and with marked environmental differences between the shady and sunny slopes. Firstly, there was a difference in illumination: the sunny slope received more sunlight throughout the day due to its orientation towards the sun, thus resulting in warmer temperatures and ample sunlight for plant growth. Vegetation on the sunny slope tended to thrive due to the abundance of sunlight. Conversely, the shady slope received less sunlight because it faced away from the sun, which led to relatively lower temperatures and potentially impacted the vegetation types and ecosystem structure in that area.

Secondly, there was a difference in precipitation: the sunny slope tended to receive more rainfall. Typically, winds blow from south to north, and the sunny slope may have been more exposed to moist air masses, thus resulting in higher precipitation levels. Higher precipitation can contribute to a lush vegetation cover and create a moist ecological environment on the sunny slope. In contrast, the shady slope may have received less rainfall due to obstructed airflow because it was more sheltered from the prevailing winds.

Different environmental factors such as sunlight exposure, moisture, and wind direction vary between different slopes. These environmental factors can impact plant growth, species competition, and interactions. Therefore, species on different slopes may exhibit distinct ecological adaptation strategies and interspecies relationships.

Influenced by both elevation and slope orientation, the sunny slopes had a notably greater abundance of dominant species than the shady slopes within the elevation bands of 1900-2200m and 2200-2500m. The species richness was also greater on the sunny slope than on the shady slope. These findings counter previous studies, such as the research conducted by Cong et al. (2023), which suggested that the number of herbaceous plant species was greater on shady slopes than on sunny slopes. However, the significantly higher positive-to-negative ratio of Spearman correlation coefficients on the shady slope might be attributed to potential adaptive differences among species that led to more negative correlations in the sunny slope environment. Some species may be better suited to the specific environmental conditions of sunny slopes, while others may not adapt as well, thereby resulting in a higher prevalence of negative correlations.

Zhang X et al. found that the species associations in a certain elevation band on the shady slopes of the Tianshan Mountains varied across regions (Zhang and Zhu, 2015). The results of Li Junling et al. indicate that the majority of herbaceous plant species pairs in the middle section of the Taihang Mountains exhibit positive correlations (Zhang et al., 2010). Jin Guili et al. discovered that on the sunny slopes, the negative associations are weak among the dominant species, with species being relatively independent, but on the shady slopes the negative associations are stronger (Jin et al., 2014). The findings of this study are somewhat inconsistent with previous research results, which demonstrates the environmental specificity of the alpine meadows in central Yunnan.

This study emphasized the importance of elevation and slope orientation in the central Yunnan region. Both are significant environmental factors that significantly influence species associations. Considering these factors not only enhances our understanding of species relationships, but it also provides deeper insights for ecosystem management and conservation. However, it is important to note that these conclusions are based on current samples and methodologies, and future research may require additional data and different approaches to confirm these findings.

### 5.2 Conclusion

1. The species diversity being higher on sunny slopes than on shady northern slopes; There are relatively few herbaceous plant species within the altitude range of 2500-2800.
2. Shady slopes had a greater number of significantly and highly significantly associated species pairs than did sunny slopes, which suggests that shady slopes have greater interspecies connectivity.
3. Species with extremely strong negative connections had a relatively high occupancy rate, which indicated a significant degree of negative interspecies association and connectivity.
4. Among dominant species pairs on both shady and sunny slopes, the number of negatively correlated species was significantly higher than that of the positively correlated ones. However, the fluctuation amplitude of the positive-to-negative ratio at different altitudes was lower on shady slopes than on sunny slopes, thus indicating an overall higher stability of the community on shady slopes.

## Data Availability

The raw data supporting the conclusions of this article will be made available by the authors, without undue reservation.
